# A Co-Rotational Based Anisotropic Elasto–Plastic Model for Geometrically Non-Linear Analysis of Fibre Reinforced Polymer Composites: Formulation and Finite Element Implementation

**DOI:** 10.3390/ma12111816

**Published:** 2019-06-04

**Authors:** Aamir Dean, Nabeel Safdar, Raimund Rolfes

**Affiliations:** Institute of Structural Analysis (ISD), Leibniz Universität Hannover, Appelstr. 9A, 30167 Hannover, Germany; n.safdar@isd.uni-hannover.de (N.S.); r.rolfes@isd.uni-hannover.de (R.R.)

**Keywords:** FRPs composites, anisotropic plasticity, co-rotational framework, finite element method (FEM)

## Abstract

Geometrical non-linearity is one of the aspects to be taken into account for accurate analysis of fibre reinforced polymers (FRPs), since large displacements and rotations may be observed in many of its structural applications such as in aircraft wings and wind turbine blades. In this paper, a co-rotational formulation and implementation of an invariant-based anisotropic plasticity model are presented for geometrically non-linear analysis of FRPs. The anisotropic constitutive equations are formulated in the format of isotropic tensors functions. The model assumes an anisotropic pressure-dependent yield function, and in addition to this, a non-associated plastic potential function in order to model realistic plastic deformations in FRPs. The formulation is then cast in the co-rotational framework to consider the geometrical non-linear effects in an efficient manner. The developed model is implemented in the commercial finite element (FE) software ABAQUS/Implicit via the means of the user-defined material subroutine (UMAT). The kinematics within the co-rotational frame is explained briefly while the important aspects regarding the numerical treatment and implementation are discussed in detail. Representative numerical examples at different scales are presented to demonstrate the applicability and robustness of the proposed development.

## 1. Introduction

Modern industry demands materials which are environmentally friendly by reducing the carbon footprint, improving safety by offering higher strengths and resistance to fatigue etc., and decreasing operational costs through virtue of fewer inspections and repairs required [1]. Recent advances in composites materials, more specifically fibre reinforced polymers (FRPs), are helping to replace traditional materials across a host of engineering applications by offering a combination of high strength to weight ratio, high stiffness, better fatigue response, reduced environmental effects, and faster manufacturing among others [2,3].

With a continuously evolving trend of shifting to composite materials, there is an ever-present need for better understanding of material behavior. Starting from simple analytical approaches to explain the material behavior, the focus gradually shifted to a more realistic and complex three-dimensional representation in the past few decades. Hence, the need to use numerical modeling came to the fore. This pronounced complexity poses a stern challenge as FRPs pose temperature, pressure, size, and rate dependencies along with the more obvious anisotropic behavior [4,5,6], and progressive failure [7,8,9].

For an accurate mechanical response prediction of FRPs along with failure behavior by means of numerical modeling techniques, the representation of anisotropy (fibre orientation) plays a fundamental role. To account for the inherently present anisotropy in the material modeling of this class of composites, generally, two main strategies are used: (i) multi-scale approach which involves in principle modeling microscopic constituents separately at corresponding scale, and (ii) macroscopic phenomenological approach which takes advantage of using extra so-called internal variables (damage, plasticity among others) to represent the characteristic non-linear material behavior under distinct loading cases. Some detailed reviews on multi-scale modeling concerning composite materials and corresponding comparisons can be found in the referenced literature [10,11,12] among many others. As the motivating thought behind numerical modeling as a virtual testing solution is efficiency along with detailed understanding of material response, the main drawback from multi-scale analysis i.e., increased computational costs goes against the soul of the objectives [13]. As a consequence, the employment of the multi-scale technique in practical engineering problems can become rather limited and impractical.

Opposed to the multi-scale approach, the anisotropic macroscopic phenomenological material modeling approach accounting for fibre orientation is promising for large engineering problems having practical real-world implications. In addition to reduced computational costs because of modeling at a single scale, only a handful number of experiments is needed for calibration and subsequent validation purposes [14,15,16]. Incorporating anisotropy into macroscopic phenomenological models can be achieved in a number of ways. One such framework is based on invariant theory [17]. In the context of this approach, the response of the material is described using scalar-valued functions through several tensorial variables such as deformation and stress tensors. To account for anisotropy, the argument list in these functions definition is extended by the so-called structural tensors which reflect the inherent symmetry of the composites material. The resulting general form of the constitutive equations is automatically invariant under coordinate transformation. For further details and a comprehensive review on the topic, the following references are useful [18,19].

In many of its structural applications (such as wind turbine blades, aircraft wings etc.), FRPs undergo large deflections and rotations, but the strains are usually within the small to moderate range because of high in-plane stiffness. Considering such behavior of FRPs, it is advantageous to use the small strain constitutive modeling which is relatively easier to handle and computationally less expensive compared to the finite strain modeling strategy (see [20]), but additionally, account for large deflections and rotations [21]. The co-rotational Lagrangian formulation provides the solution, where the idea is to decompose the motion of the body into rigid body motions i.e., deflections and rotations, and pure deformations. It has been mostly employed for beam and shell formulations for isotropic materials [22,23,24,25] as such beam and shell elements are used for applications within small deformations, but it is not limited to that. Rather, it can be employed in any finite element (FE) formulation where the basic assumption of small strains and arbitrary rotations is fulfilled as highlighted in the references [26,27,28].

The fundamental concept in the co-rotational formulation is the split of motions of a continuum body into two steps. In the first step, the rigid translations and rotations of the undeformed body are considered. The rigid translations are defined by displacements expressed in the global frame of reference. The rigid rotations, defined by an orthogonal rotation matrix, defines the orientation of local frame in the deformed configuration. In the second step, the local deformation of the body with respect to the local frame of reference is considered. This approach predates finite element methods (FEM) by over a century. Recently, the idea has successfully found application in FEM [25,28]. The pure deformation part of the displacement field, obtained by subtracting rigid body motions from the total displacement field, tends to be small when the incremental motion is sufficiently small. This argumentation is the basis for the infinitesimal magnitude of strains in the rotated frame. In a spatially discretized domain such as in FEM, this decomposition of the motions of the body is achieved by defining a local co-rotational frame for each discretized element. This local frame does not deform but rather translates and rotates with the element. The pure deformational part of the motion measured with respect to this local frame is small. Hence, the discrete gradients of the deformational displacement field in the local frame are of the order of small strains [29]. This key concept helps to simplify the updated Lagrangian formulation to the co-rotational formulation. For more details, the reader is referred to [24,30].

In this contribution, an invariant-based anisotropic elasto–plastic model is formulated and implemented within the co-rotational framework for its application in geometrically non-linear analysis of FRPs. The anisotropic constitutive equations are represented in the form of isotropic tensor functions. Accordingly, an anisotropic pressure-dependent yield surface is introduced along with a non-associative plastic potential function to account for the non-linear inelastic material behavior [31]. Employing the non-associative flow rule allows for modeling realistic plastic deformation compared to associative plasticity, especially with regard to contractility/dilatancy effects resulting in different behavior under compression and tension as it is observed in composites. The model is then cast into the co-rotational framework so that the geometrical non-linear effects (large deflections and rotations) can be included. It is to be noted that although the strains are assumed to be within small to moderate range, they are not exactly the small strains obtained using linear deformation theory [24,30]. Afterward, the computational aspects corresponding to the algorithmic treatment of the proposed model and its numerical implementation are detailed. Novel closed form expressions, necessary for a consistent FE implementation, are also derived.

For the sake of transparency, this paper focuses on the extension of the geometrically linear plasticity model presented in [15] for unidirectional (UD) FRPs, to take into account the geometrical non-linear effects due to large displacements and rotations. In comparison to the constitutive model in [15], modifications to the yield and plastic potential function definitions are proposed. These modifications allow for an easier calibration of the yield surface and plastic potential function with the experimental data. In this regard, explicit expressions for the yield surface and plastic potential parameters are provided. From the computational side, herein an explicit expression for the algorithmic consistent tangent moduli is derived.

The paper is organized into the following sections: Section 2 discusses the constitutive formulation of the invariant-based anisotropic elasto–plastic material model within the co-rotational framework in detail. Section 3 details the numerical treatment of the proposed model including the FE implementation procedure in the commercial software ABAQUS 2017. Thereafter, some numerical results are presented in Section 4 to highlight the validity and range of application of the proposed formulation. Finally, the main conclusions of the current contribution are drawn in Section 5.

## 2. Constitutive Formulation

This section presents the constitutive formulation of the anisotropic invariant-based model for FRPs. It is to be noted that the constitutive model proposed here is a modification of the one presented in [15]. These modifications include a new form of the yield and plastic potential functions. Nevertheless, for the sake of clarity and completeness, the constitutive formulation is provided in detail.

It should be noted that the constitutive equations are formulated with respect to the co-rotational frame.

### 2.1. Transversely Isotropic Free-Energy Definition

From the modeling standpoint, the anisotropic mechanical response admits a tensor-based representation through the definition of a second order structural tensor A in the rotated frame. The structural tensor represents the anisotropic material inherent structure and is defined as:

(1) A:=a⊗a,

where a identifies the fibre orientation vector in the rotated frame.

Based on the hypothesis of the flow theory of plasticity, the total strain tensor ε is additively decomposed into elastic $\varepsilon^{e}$ and plastic $\varepsilon^{p}$ counterparts as follows:

(2) $$\varepsilon =\varepsilon^{e}+\varepsilon^{p}.$$

For the constitute formulation, the existence of a Helmholtz free-energy function, $\Psi \left(\varepsilon^{e},A,\varpi \right)$ is assumed. This free-energy is a function of the elastic strain $\varepsilon^{e}$, the structural tensor A, and the internal variable set ϖ that accounts for the inelastic material response along the deformation process:

(3) $$\Psi \left(\varepsilon^{e},A,\varpi \right)=\frac{1}{2}\varepsilon^{e}:C^{e}:\varepsilon^{e}+\Psi^{\mathrm{hard}}\left(A,\varpi \right),$$

where $C^{e}$ is the constitutive elastic tensor and $\Psi^{\mathrm{hard}}\left(A,\varpi \right)$ is the hardening part of the free-energy function due to plastic effects.

Having on hand the free energy function definition, the constitutive stress tensor σ is obtained as the first derivative of the free energy function with respect to the elastic strain tensor, while the elastic constitutive operator $C^{e}$ is defined as the second derivative of the free energy with respect to elastic strain tensor:

(4) $$\sigma :=\frac{\partial \Psi }{\partial \varepsilon^{e}}=C^{e}:\varepsilon^{e},$$

For transversely isotropic materials, the constitutive transversely isotropic elasticity tensor is represented as follows:

(5) $$C^{e}:=\frac{\partial^{2}\Psi }{\partial \varepsilon^{e}\partial \varepsilon^{e}}=\lambda 1⊗1+2\mu_{T}I+\alpha \left(1⊗A+A⊗1\right)+2\left(\mu_{L}-\mu_{T}\right)I_{A}+\beta A⊗A,$$

where I refers to the fourth-order identity tensor, whereas $I_{A}=A_{im}I_{jmkl}+A_{jm}I_{mikl}$, and λ, α, β, $\mu_{T}$ and $\mu_{L}$ are the elastic constants. Their definition and relationship to the engineering constants are given in [4].

### 2.2. Thermodynamics Considerations

The constitutive equations are restricted by the second-law of thermodynamics in the form of the Clausius–Duhem inequality. Under the assumption of isothermal deformations, this inequality reads the following for internal energy dissipation $D_{\mathrm{int}}$:

(6) $$D_{\mathrm{int}}=\sigma :\dot{\varepsilon }-\dot{\Psi }\geq 0.$$

Recalling the previous definitions, the restriction over the internal dissipation reads:

(7) $$D_{\mathrm{int}}=\sigma :{\dot{\varepsilon }}^{p}+\Gamma *\dot{\varpi }\geq 0.$$

where Γ denotes the so-called hardening force and * stands for any arbitrary product.

### 2.3. Yield Function

The elastic domain E, assuming the maximum dissipation principle, is defined as:

(8) $$E=\{\left(\varpi ,{\bar{\varepsilon }}^{p}\right)|F\left(\sigma ,A,{\bar{\varepsilon }}^{p}\right)\leq 0\},$$

where ${\bar{\varepsilon }}^{p}$ identifies the equivalent plastic strain. The definition of the equivalent plastic strain in the present formulation is given by:

(9) $${\bar{\varepsilon }}^{p}:=\sqrt{\frac{1}{2}}∥\varepsilon^{p}∥.$$

The construction of a transversely isotropic yield surface $F(\sigma ,A,{\bar{\varepsilon }}^{p})$, which accounts for the pressure-dependency and plastic-inextensibility in FRPs along the fibre direction yields:

(10) $$F\left(\sigma ,A,{\bar{\varepsilon }}^{p}\right)=\zeta_{1}I_{1}+\zeta_{2}I_{2}+\zeta_{3}I_{3}+\zeta_{4}I_{3}^{2}-1\leq 0,$$

$I_{i}$ (i=1,3) are the stress invariants which symbolize the integrity basis of an isotropic tensor function representing a transversely isotropic response:

(11) $$I_{1}=\frac{1}{2}{\left(\mathrm{tr}\left[\sigma^{\mathrm{pind}}\right]\right)}^{2}-\mathrm{tr}\left[A{\left(\sigma^{pind}\right)}^{2}\right];\;I_{2}=\mathrm{tr}\left[A{\left(\sigma^{\mathrm{pind}}\right)}^{2}\right];\;I_{3}=\mathrm{tr}\left[\sigma \right]-\mathrm{tr}\left[A\left(\sigma \right)\right],$$

where $\sigma^{\mathrm{pind}}$ is the plasticity-inducing stress:

(12) $$\sigma^{\mathrm{pind}}:=\sigma -\frac{1}{2}\left(\mathrm{tr}\left[\sigma \right]-a\sigma a\right)1+\frac{1}{2}\left(\mathrm{tr}\left[\sigma \right]-3a\sigma a\right)A,$$

Here, $\zeta_{i}\left({\bar{\varepsilon }}^{p}\right)$ (i=1,4) refers to four yield parameters which together with their corresponding invariants represent different loading states.

A compact representation of the yield function takes the form:

(13) $$F\left(\sigma ,A,{\bar{\varepsilon }}^{p}\right)=\frac{1}{2}\sigma :K:\sigma +L:\sigma -1\leq 0,$$

where

(14) $$K:=\zeta_{1}P^{\mathrm{pind}}+\left(\zeta_{2}-\zeta_{1}\right)P_{A}^{\mathrm{pind}}+2\zeta_{4}\left(1-A\right)⊗\left(1-A\right);\;L:=\zeta_{3}\left(1-A\right),$$

where the operators $P^{\mathrm{pind}}$ and $P_{A}^{ind}$ are defined as:

(15) $$P^{\mathrm{pind}}=I-\frac{1}{2}\left(1⊗1\right)+\frac{1}{2}\left(A⊗1+1⊗A\right)-\frac{3}{2}\left(A⊗A\right);\;P_{A}^{\mathrm{pind}}:=P_{Aijkl}^{\mathrm{pind}}=A_{im}P_{mjkl}^{\mathrm{pind}}+A_{mj}P_{imkl}^{\mathrm{pind}}.$$

In comparison to the six-parameter yield surface definition in [15], herein a four-parameter yield surface is proposed. The herein proposal allows for an easier calibration of the yield surface and reduces the experimental effort. Nevertheless, the six-parameter yield function definition regards a better description of biaxial stress states which is crucial for accurate modeling of FRPs undergoing high hydrostatic pressures. This is achieved in [15] via the case differentiation concerning the invariant $I_{3}$ based on its sign.

### 2.4. Plastic Potential Function

To predict realistic plastic deformations, a non-associative flow rule is assumed. The construction of a non-associative transversely isotropic plastic potential function G(σ,A) yields:

(16) $$G\left(\sigma ,A\right)=\varsigma_{1}I_{1}+\varsigma_{2}I_{2}+\varsigma_{3}I_{3}^{2}-1,$$

where $\varsigma_{i}$ (i=1,3) denotes the plastic potential parameters. A condensed expression of the plastic flow potential is given by:

(17) $$G\left(\sigma ,A\right)=\frac{1}{2}\sigma :M:\sigma -1\leq 0,$$

where the fourth-order tensor M is expressed as:

(18) $$M:=\varsigma_{1}P^{\mathrm{pind}}+\left(\varsigma_{2}-\varsigma_{1}\right)P_{A}^{\mathrm{pind}}+2\varsigma_{3}\left(1-A\right)⊗\left(1-A\right).$$

### 2.5. Evolution Equations

The evolution equations of the internal variables ($\varepsilon^{p}$ and ϖ) are expressed as follows:

(19) $${\dot{\varepsilon }}^{p}=\dot{\gamma }\frac{\partial G(\sigma ,A,{\bar{\varepsilon }}^{p})}{\partial \sigma }=\dot{\gamma }n_{G}=\dot{\gamma }M:\sigma \;\mathrm{with}\;n_{G}=M:\sigma ,$$

(20) $$\dot{\varpi }=\dot{\gamma }\frac{\partial G(\sigma ,A,{\bar{\varepsilon }}^{p})}{\partial \Gamma },$$

where γ represents the so-called plastic multiplier.

As customary, the Kuhn–Tucker loading/unloading conditions are defined by:

(21) $$\dot{\gamma }\geq 0;\;F\left(\sigma ,A,{\bar{\varepsilon }}^{p}\right)\leq 0;\;\dot{\gamma }F\left(\sigma ,A,{\bar{\varepsilon }}^{p}\right)=0,$$

and the consistency condition as:

(22) $$\dot{\gamma }\dot{F}\left(\sigma ,A,{\bar{\varepsilon }}^{p}\right)=0.$$

### 2.6. Parameter Identification

In addition to the elastic material constants, the yield function parameters $\zeta_{i}$ (i=1,4) and the plastic potential parameters $\varsigma_{i}$ (i=1,3) are to be determined.

The parameters $\zeta_{i}$ (i=1,4) control the size and shape of the elastic domain E as a function of the equivalent plastic strain variable ${\bar{\varepsilon }}^{p}$. For each parameter, the relation $\zeta_{i}\left({\bar{\varepsilon }}^{p}\right)$ is determined from an independent experiment, thus a total of four different experiments is required for calibration. For instance, the following four experiments can be employed for calibration: (i) in-plane shear test, (ii) transverse shear test, (iii) uniaxial transverse tension test, and (iv) uniaxial transverse compression test. The corresponding yield stress states are denoted as $\sigma_{is}^{y},\sigma_{ts}^{y},\sigma_{tt}^{y}$, and $\sigma_{tc}^{y}$, respectively. Similar to the procedure in [15], the four parameters $\zeta_{i}\left(\sigma_{is}^{y},\sigma_{ts}^{y},\sigma_{tt}^{y},\sigma_{tc}^{y}\right)$ (i=1,4) can then be obtained by entering the stress states from each experiments above in Equation (10) and setting the yield function state to yielding i.e., F=0. Accordingly, the coefficients $\zeta_{i}$ (i=1,4) are explicitly given in the following.

From the in-plane shear test the first coefficient $\zeta_{1}$ is expressed as:

(23) $$\zeta_{1}=\frac{1}{{\sigma_{ts}^{y}}^{2}},$$

and from the transverse shear test the second coefficient $\zeta_{2}$ is given by:

(24) $$\zeta_{2}=\frac{1}{{\sigma_{is}^{y}}^{2}}.$$

The third coefficient $\zeta_{3}$ controls the tension-compression yield asymmetry and therefore is expressed in terms of the uniaxial transverse tension and uniaxial transverse compression tests as:

(25) $$\zeta_{3}\;=-\frac{1}{\sigma_{tc}^{y}}+\frac{1}{\sigma_{tt}^{y}},$$

Lastly, the coefficient $\zeta_{4}$ is associated with transverse loading, hence is expressed as:

(26) $$\zeta_{4}\;=-\frac{1}{4{\sigma_{ts}^{y}}^{2}}+\frac{1}{\sigma_{tc}^{y}\sigma_{tt}^{y}}.$$

To comply with the maximum dissipation principle, the convexity of the yield surface must be insured. This imposes the following restrictions to the relations $\zeta_{i}\left({\bar{\varepsilon }}^{p}\right)$ (i=1,4) which must hold for any ${\bar{\varepsilon }}^{p}$:

(27) $$\sigma_{tt}^{y}\leq \frac{4{\sigma_{ts}^{y}}^{2}}{\sigma_{tc}^{y}}.$$

Similary, the parameters $\varsigma_{i}$ (i=1,3) control the size and shape of the plastic potential surface. However, one of these parameters is a scaling parameter and can be set to any value since the size of the plastic potential has no inherent physical meaning. Accordingly, there are only two remaining parameters to be determined and to associate with experimental data. In the present case, $\varsigma_{1}$ is arbitrarily set to unity.

As mentioned above, the motive behind adopting a non-associative plasticity scheme is to model realistic plastic deformation behavior as compared to associative plasticity. Accordingly, the parameters $\varsigma_{i}$ (i=2,3) are used to enforce certain plastic Poisson’s ratios $\nu_{23}^{p}=\varepsilon_{22}^{p}/\varepsilon_{33}^{p}$ and plastic distortion behavior through the relation $\mu_{12}^{p}=\varepsilon_{12}^{p}/\varepsilon_{23}^{p}$:

(28) $$\varsigma_{1}=1,$$

(29) $$\varsigma_{2}=\mu_{12}^{p},$$

(30) $$\varsigma_{3}=\frac{-1+\nu_{23}^{p}}{4(1+\nu_{23}^{p})}.$$

Similarly, for the plastic potential function G, the following must hold:

(31) $$\mu_{12}^{p}\geq 0\wedge -\frac{-1+\nu_{23}^{p}}{4(1+\nu_{23}^{p})}\geq 0.$$

In contrast to the time-consuming iterative procedure presented in [15] for the determination of the plastic potential parameters, herein explicit expressions for the parameters are provided.

## 3. Numerical Treatment

In this section, the numerical treatment of the constitutive model proposed in Section 2 is discussed.

The construction of a numerical scheme for the solution of the initial boundary value problem (IBVP) associated with the current elasto–plastic model involved two main aspects [32]. The first concerned the local (at the Gauss point in FE context) integration of the evolution equations. The second regarded the employment of the result stemming from the previous step in the constitutive block of the weak formulation of the balance of linear momentum, which was discretized in space by means of FEM and solved by means of a standard incremental-iterative Newton–Raphson scheme.

It should be noted that all quantities presented in this section are computed in the rotated frame $B_{n+1}^{\mathrm{rot}}$.

### 3.1. Numerical Integration: General Return Mapping Algorithm

For a prescribed motion of an arbitrary body, let us consider the time interval $\left[t_{n},t_{n+1}^{\left(i\right)}\right]$, with $t\in R_{+}$, where $t_{n}$ identifies the previous converged time step and $t_{n+1}^{\left(i\right)}$ denotes the current prospective time step at the global Newton–Raphson iteration *i*. The strain rate within the time step were given by:

(32) $$\dot{\varepsilon }=\frac{\varepsilon_{n+1}-\varepsilon_{n}}{\Delta t};\;\;\mathrm{with}\;\;\Delta t=t_{n+1}-t_{n}.$$

To simplify the notation, the superscript *i* is omitted.

The internal variables $\varepsilon_{n}^{p}$, ${\bar{\varepsilon }}_{n}^{p}$ and $\varpi_{n}$, and the prospective total strain $\varepsilon_{n+1}$ are assumed to be available. Then, the elasto–plastic constitutive boundary value problem at the material (Gauss) point level is stated as follows:

Given: $\varepsilon_{n}^{p}$, ${\bar{\varepsilon }}_{n}^{p}$, $\varpi_{n}$, and $\varepsilon_{n+1}$,

Find: $\varepsilon_{n+1}^{p}$, ${\bar{\varepsilon }}_{n+1}^{p}$, and $\varpi_{n+1}$ at the end of the time interval $\left[t_{n},t_{n+1}\right]$,

Such that:

(33) $${\dot{\varepsilon }}^{e}=\dot{\varepsilon }-\dot{\gamma }n_{G};\;{\dot{\bar{\varepsilon }}}^{p}=\dot{\gamma }\sqrt{\frac{1}{2}}∥n_{G}∥,$$

with

(34) $$\dot{\gamma }\geq 0;\;F\left(\sigma ,A,{\bar{\varepsilon }}^{p}\right)\leq 0;\;\dot{\gamma }F\left(\sigma ,A,{\bar{\varepsilon }}^{p}\right)=0.$$

The central point for the local integration of the model is the adoption of the backward-Euler (fully implicit, first-order accurate and unconditionally stable) integration scheme. Accordingly, the discrete version of the rate expressions given in Equations (32) and (33) within the interval $\left[t_{n},t_{n+1}\right]$ are obtained as follows:

(35) $$\varepsilon_{n+1}^{e}=\varepsilon_{n}^{e}+\Delta \varepsilon -\gamma_{n+1}n_{G,n+1};\;{\bar{\varepsilon }}_{n+1}^{p}={\bar{\varepsilon }}_{n}^{p}+\gamma_{n+1}\sqrt{\frac{1}{2}}∥n_{G,n+1}∥,$$

with

(36) $$\gamma_{n+1}\geq 0;\;F\left(\sigma_{n+1},A,{\bar{\varepsilon }}_{n+1}^{p}\right)\leq 0;\;\gamma_{n+1}F\left(\sigma_{n+1},A,{\bar{\varepsilon }}_{n+1}^{p}\right)=0,$$

where $\Delta \varepsilon =\varepsilon_{n+1}-\varepsilon_{n}$.

Next, the classical two-step predictor-corrector procedure [33] is applied. The first step concerns the computation of the predictor elastic trial step as follows:

(37) $$\varepsilon_{n+1}^{e,\mathrm{trial}}=\varepsilon_{n}^{e}+\Delta \varepsilon \;\;\mathrm{and}\;\;{\bar{\varepsilon }}_{n+1}^{p,\mathrm{trial}}={\bar{\varepsilon }}_{n}^{p},$$

(38) $$\sigma_{n+1}^{\mathrm{trial}}=C_{e}:\varepsilon_{n+1}^{e,\mathrm{trial}}.$$

The corresponding trial yield function is given by:

(39) $$F\left(\sigma_{n+1}^{\mathrm{trial}},A,{\bar{\varepsilon }}_{n}^{p}\right)=\frac{1}{2}\sigma_{n+1}^{\mathrm{trial}}:K^{\mathrm{trial}}:\sigma_{n+1}^{\mathrm{trial}}+L^{\mathrm{trial}}:\sigma_{n+1}^{\mathrm{trial}}-1,$$

where the operators $K^{\mathrm{trial}}$ and $L^{\mathrm{trial}}$ are function of the trial equivalent plastic strain ${\bar{\varepsilon }}_{n+1}^{p,\mathrm{trial}}$.

As customary, if the elastic trial state lies within the elastic domain i.e., $F(\sigma_{n+1}^{\mathrm{trial}},A,{\bar{\varepsilon }}_{n}^{p})<0$, then the solution is elastic with $\gamma_{n+1}=0$ and the trial step is accepted as the correct solution. Otherwise, the solution is plastic with $\gamma_{n+1}>0$ and is obtained via the plastic corrector step fulfilling the constraint:

(40) $$F_{n+1}\left(\sigma_{n+1},A,{\bar{\varepsilon }}_{n+1}^{p}\right)\overset{!}{=}0.$$

Based on this, the computation of the plastic multiplier $\gamma_{n+1}$ follows the procedure outlined in Algorithm 1.

**Algorithm 1** Plastic corrector step: algorithmic computation of the plastic multiplier and update of the internal variables.
Compute $\varepsilon_{n+1}^{e,\mathrm{trial}}=\varepsilon_{n+1}^{e}+\gamma_{n+1}n_{G,n+1}$.Substitute $n_{G,n+1}=M:\sigma_{n+1}$→$\varepsilon_{n+1}^{e,\mathrm{trial}}=\varepsilon_{n+1}^{e}+\gamma_{n+1}M:\sigma_{n+1}$.Compute $C^{e}:\varepsilon_{n+1}^{e,\mathrm{trial}}=C^{e}:\varepsilon_{n+1}^{e}+\gamma_{n+1}C^{e}:M:\sigma_{n+1}$.Identify $\sigma_{n+1}^{\mathrm{trial}}=\sigma_{n+1}+\gamma_{n+1}C^{e}:M:\sigma_{n+1}$.Compute $\sigma_{n+1}={\left[I+\gamma_{n+1}C^{e}:M\right]}^{-1}:\sigma_{n+1}^{\mathrm{trial}}=H:\sigma_{n+1}^{\mathrm{trial}}$; with $H={\left[I+\gamma_{n+1}C^{e}:M\right]}^{-1}$.Solve the equation to determine the consistency parameter $\gamma_{n+1}$ via local iterative process (local Newton–Raphson index denoted by the superscript *k*).
(a)Set k=0 and the initial values $\left(\sigma_{n+1}^{\left(k=0\right)}=\sigma_{n+1}^{\mathrm{trial}},{\bar{\varepsilon }}_{n+1}^{p,(k=0)}={\bar{\varepsilon }}_{n}^{p},\gamma_{n+1}^{\left(k=0\right)}=0\right)$. (b)Compute $F^{\left(k\right)}\left(\sigma_{n+1},A,{\bar{\varepsilon }}_{n+1}^{p}\right)$. (c)IF $F^{\left(k\right)}\left(\sigma_{n+1},A,{\bar{\varepsilon }}_{n+1}^{p}\right)\leq \mathrm{TOL}$ GOTO 7, ELSE (d)Set residual for local Newton-Rapshon iteration $R_{n+1}^{\left(k\right)}=F^{\left(k\right)}\left(\sigma_{n+1},A,{\bar{\varepsilon }}_{n+1}^{p}\right)$. (e)Perform linearization of $R_{n+1}^{\left(k\right)}$: $\hat{L}\left[R_{n+1}^{\left(k\right)}\right]≃R_{n+1}^{\left(k\right)}+\Delta \gamma^{\left(k\right)}\left[\frac{\partial F_{n+1}^{\left(k\right)}}{\partial \sigma_{n+1}^{\left(k\right)}}:\frac{\partial \sigma_{n+1}^{\left(k\right)}}{\partial \gamma_{n+1}^{\left(k\right)}}+\frac{\partial F_{n+1}^{\left(k\right)}}{\partial K_{n+1}^{\left(k\right)}}\cdot \cdot \cdot \cdot \frac{\partial K_{n+1}^{\left(k\right)}}{\partial \gamma_{n+1}^{\left(k\right)}}+\frac{\partial F_{n+1}^{\left(k\right)}}{\partial L_{n+1}^{\left(k\right)}}:\frac{\partial L_{n+1}^{\left(k\right)}}{\partial \gamma_{n+1}^{\left(k\right)}}\right]=0$. (f)Compute $\Delta \gamma^{\left(k\right)}=\frac{-R_{n+1}^{\left(k\right)}}{\frac{\partial F_{n+1}^{\left(k\right)}}{\partial \sigma_{n+1}^{\left(k\right)}}:\frac{\partial \sigma_{n+1}^{\left(k\right)}}{\partial \gamma_{n+1}^{\left(k\right)}}+\frac{\partial F_{n+1}^{\left(k\right)}}{\partial K_{n+1}^{\left(k\right)}}\cdot \cdot \cdot \cdot \frac{\partial K_{n+1}^{\left(k\right)}}{\partial \gamma_{n+1}^{\left(k\right)}}+\frac{\partial F_{n+1}^{\left(k\right)}}{\partial L_{n+1}^{\left(k\right)}}:\frac{\partial L_{n+1}^{\left(k\right)}}{\partial \gamma_{n+1}^{\left(k\right)}}}$. (g)Correct $\gamma_{n+1}^{\left(k+1\right)}=\gamma_{n+1}^{\left(k\right)}+\Delta \gamma^{\left(k\right)}$. (h)k←k+1 GOTO (b)  Update the internal variables $\sigma_{n+1}=\sigma_{n+1}^{\left(k\right)}$, ${\bar{\varepsilon }}_{n+1}^{p}={\bar{\varepsilon }}_{n+1}^{p,(k)}$, $\varepsilon_{n+1}^{p}=\varepsilon_{n+1}^{p,(k)}$.Compute algorithmic tangent operator, see Section 3.2.


The expressions required for the computation of Algorithm 1 are provided in the following. The first term within the denominator of the linearization $\frac{\partial F_{n+1}^{\left(k\right)}}{\partial \sigma_{n+1}^{\left(k\right)}}$ takes the form:

(41) $$\frac{\partial F_{n+1}^{\left(k\right)}}{\partial \sigma_{n+1}^{\left(k\right)}}=K_{n+1}^{\left(k\right)}:\sigma_{n+1}^{\left(k\right)}+L_{n+1}^{\left(k\right)},$$

where

(42) $$\sigma_{n+1}^{\left(k\right)}=H_{n+1}^{\left(k\right)}:\sigma_{n+1}^{\mathrm{trial}},$$

and

(43) $${\bar{\varepsilon }}_{n+1}^{p,(k)}={\bar{\varepsilon }}_{n}^{p,(k)}+\gamma_{n+1}^{\left(k\right)}\sqrt{\frac{1}{2}}∥M_{n+1}:\sigma_{n+1}^{\left(k\right)}∥.$$

The second term $\frac{\partial \sigma_{n+1}^{\left(k\right)}}{\partial \gamma_{n+1}^{\left(k\right)}}$ is expressed as:

(44) $$\frac{\partial \sigma_{n+1}^{\left(k\right)}}{\partial \gamma_{n+1}}=-H_{n+1}^{\left(k\right)}:\left[\left(C_{e}:M_{n+1}\right):\sigma_{n+1}^{\left(k\right)}\right].$$

The third and fifth terms take the form, respectively:

(45) $$\frac{\partial F_{n+1}^{\left(k\right)}}{\partial K_{n+1}^{\left(k\right)}}=\frac{1}{2}\left[\sigma_{n+1}^{\left(k\right)}⊗\sigma_{n+1}^{\left(k\right)}\right];\;\frac{\partial F_{n+1}^{\left(k\right)}}{\partial L_{n+1}^{\left(k\right)}}=\sigma_{n+1}^{\left(k\right)}.$$

The fourth term reads:

(46) $$\frac{\partial K_{n+1}^{\left(k\right)}}{\partial \gamma_{n+1}^{\left(k\right)}}=\frac{\partial K_{n+1}^{\left(k\right)}}{\partial {\bar{\varepsilon }}_{n+1}^{p,(k)}}\frac{\partial {\bar{\varepsilon }}_{n+1}^{p,(k)}}{\partial \gamma_{n+1}^{\left(k\right)}}.$$

The term $\frac{\partial K_{n+1}^{\left(k\right)}}{\partial {\bar{\varepsilon }}_{n+1}^{p,(k)}}$ in Equation (46) is expressed as:

(47) $$\frac{\partial K_{n+1}^{\left(k\right)}}{\partial {\bar{\varepsilon }}_{n+1}^{p,(k)}}=\sum_{i=1,2,4}\frac{\partial K_{n+1}^{\left(k\right)}}{\partial \zeta_{i}^{\left(k\right)}}\frac{\partial \zeta_{i}^{\left(k\right)}}{\partial {\bar{\varepsilon }}_{n+1}^{p,(k)}}=\left[P^{\mathrm{ind}}-P_{A}^{\mathrm{ind}}\right]\frac{\partial \zeta_{1}^{\left(k\right)}}{\partial {\bar{\varepsilon }}_{n+1}^{p,(k)}}+P_{A}^{\mathrm{ind}}\frac{\partial \zeta_{2}^{\left(k\right)}}{\partial {\bar{\varepsilon }}_{n+1}^{p,(k)}}+2\left(1-A\right)⊗\left(1-A\right)\frac{\partial \zeta_{4}^{\left(k\right)}}{\partial {\bar{\varepsilon }}_{n+1}^{p,(k)}},$$

where

(48) $$\frac{\partial {\bar{\varepsilon }}_{n+1}^{p,(k)}}{\partial \gamma_{n+1}^{\left(k\right)}}=\sqrt{\frac{1}{2}}∥M_{n+1}:\sigma_{n+1}^{\left(k\right)}∥+\gamma_{n+1}^{\left(k\right)}\sqrt{\frac{1}{2}}\left[\frac{\left[M_{n+1}:\sigma_{n+1}^{\left(k\right)}\right]:M_{n+1}}{∥M_{n+1}:\sigma_{n+1}^{\left(k\right)}∥}\right]:\frac{\partial \sigma_{n+1}^{\left(k\right)}}{\partial \gamma_{n+1}^{\left(k\right)}}.$$

Lastly, the sixth terms $\frac{\partial L_{n+1}^{\left(k\right)}}{\partial \gamma_{n+1}^{\left(k\right)}}$ takes the form:

(49) $$\frac{\partial L_{n+1}^{\left(k\right)}}{\partial \gamma_{n+1}^{\left(k\right)}}=\frac{\partial L_{n+1}^{\left(k\right)}\left(\zeta_{3}^{\left(k\right)}\right)}{\partial {\bar{\varepsilon }}_{n+1}^{p,(k)}}\frac{\partial {\bar{\varepsilon }}_{n+1}^{p,(k)}}{\partial \gamma_{n+1}^{\left(k\right)}}.$$

where

(50) $$\frac{\partial L_{n+1}^{\left(k\right)}}{\partial {\bar{\varepsilon }}_{n+1}^{p,(k)}}=\frac{\partial L_{n+1}^{\left(k\right)}}{\partial \zeta_{3}^{\left(k\right)}}\frac{\partial \zeta_{3}^{\left(k\right)}}{\partial {\bar{\varepsilon }}_{n+1}^{p,(k)}}=\left(1-A\right)\frac{\partial \zeta_{3}^{\left(k\right)}}{\partial {\bar{\varepsilon }}_{n+1}^{p,(k)}}.$$

### 3.2. Algorithmic Consistent Tangent Moduli

For the solution of the non-linear FE equations (discretized weak form of the balance of linear momentum) on a global level, the incremental-iterative Newton–Raphson scheme is used [32]. Therein, in order to obtain a quadratic convergence, the computation of the algorithmic consistent tangent moduli is required, i.e., consistent with the chosen algorithmic time integration scheme.

The form, $d\sigma_{n+1}=C_{n+1}^{\mathrm{ep}}:d\varepsilon_{n+1}$ describes the sensitivity of the stress with respect to an infinitesimal increment in the strain at time $t_{n+1}$ When the local integration algorithm described has converged is looked for.

The starting point to derive the algorithmic consistent tangent moduli is forming an expression for the infinitesimal increment of the total stress at time $t_{n+1}$. Using the relation $\sigma_{n+1}=H:\sigma_{n+1}^{\mathrm{trial}}$ in Algorithm 1, the increment of the total stress reads:

(51) $$d\sigma_{n+1}=H_{n+1}:\left[C^{e}:d\varepsilon_{n+1}-d\gamma_{n+1}\left[C^{e}:M_{n+1}\right]:\sigma_{n+1}\right],$$

Next, an explicit expression for the differential of the plastic multiplier $d\gamma_{n+1}$ is to be obtained. This is achieved through the consistency condition given in Equation (22). In case of plastic loading i.e., $\gamma_{n+1}\geq 0$, ${\dot{F}}_{n+1}=0$ and therefore $dF_{n+1}=0$. Accordingly from the condition $dF_{n+1}=0$, $d\gamma_{n+1}$ is obtained as:

(52) $$d\gamma_{n+1}=-\frac{\frac{\partial F_{n+1}^{*}}{\partial \varepsilon_{n+1}}:d\varepsilon_{n+1}}{\frac{\partial F_{n+1}^{*}}{\partial \gamma_{n+1}}},$$

where the term $\frac{\partial F_{n+1}^{*}}{\partial \varepsilon_{n+1}}$ reads:

(53) $$\frac{\partial F_{n+1}^{*}}{\partial \varepsilon_{n+1}}=\frac{\partial F_{n+1}}{\partial \sigma_{n+1}}:\left[H_{n+1}:C^{e}\right]+\frac{\partial F_{n+1}}{\partial {\bar{\varepsilon }}_{n+1}^{p}}\sqrt{\frac{1}{2}}\frac{\gamma_{n+1}}{∥M_{n+1}:\sigma_{n+1}∥}\left[\left[M_{n+1}:\sigma_{n+1}\right]:M_{n+1}\right]:\left[H_{n+1}:C^{e}\right],$$

where

(54) $$\frac{\partial F_{n+1}}{\partial {\bar{\varepsilon }}_{n+1}^{p}}=\frac{\partial F_{n+1}}{\partial K_{n+1}}\cdot \cdot \cdot \cdot \frac{\partial K_{n+1}}{\partial {\bar{\varepsilon }}_{n+1}^{p}}+\frac{\partial F_{n+1}}{\partial L_{n+1}}:\frac{\partial L_{n+1}}{\partial {\bar{\varepsilon }}_{n+1}^{p}}.$$

The term $\frac{\partial F_{n+1}^{*}}{\partial \gamma_{n+1}}$ takes the form:

(55) $$\begin{array}{c} \frac{\partial F_{n+1}^{*}}{\partial \gamma_{n+1}}=-\frac{\partial F_{n+1}}{\partial \sigma_{n+1}}:\left[H_{n+1}:\left[\left[C^{e}:M_{n+1}\right]:\sigma_{n+1}\right]\right]\\ +\frac{\partial F_{n+1}}{\partial {\bar{\varepsilon }}_{n+1}^{p}}\sqrt{\frac{1}{2}}\left[∥M_{n+1}:\sigma_{n+1}∥-\frac{\gamma_{n+1}}{∥M_{n+1}:\sigma_{n+1}∥}\left[\left[M_{n+1}:\sigma_{n+1}\right]:M_{n+1}\right]:\left[H_{n+1}:\left[\left[C^{e}:M_{n+1}\right]:\sigma_{n+1}\right]\right]\right]. \end{array}$$

Finally, by substituting the expression for $d\gamma_{n+1}$ in Equation (51), the algorithmic consistent tangent moduli $C_{n+1}^{ep}$ is given by:

(56) $$C_{n+1}^{ep}=\frac{\partial \sigma_{n+1}}{\partial \varepsilon_{n+1}}=H_{n+1}:\left[C^{e}+\left[\left[C^{e}:M_{n+1}\right]:\sigma_{n+1}\right]⊗\frac{\frac{\partial F_{n+1}^{*}}{\partial \varepsilon_{n+1}}}{\frac{\partial F_{n+1}^{*}}{\partial \gamma_{n+1}}}\right].$$

### 3.3. FE Implementation in ABAQUS

Herein, the numerical implementation of the model in the general purpose FE code ABAQUS/Implicit via the user-defined subroutine UMAT is described.

During the global computation, the subroutine UMAT was called at all material calculation points of elements for which the material definition includes a user-defined material behavior. The subroutine must update the stress (σ) and solution-dependent state (internal) variables ($\varepsilon^{p}$ and ${\bar{\varepsilon }}^{p}$) to their values at the end of the increment for which it is called and also provide the material Jacobian matrix ($C^{ep}$), see [34].

The incremental strain (Δε) and the total strain ($\varepsilon_{n+1}$) in the rotated frame were passed in by the UMAT and their components are rotated to account for rigid body motion in the increment before UMAT was called.

The stress at the beginning of the increment ($\sigma_{n}$) is also passed in. The stress is already rotated to account for rigid body motion in the increment and must be updated in the routine to be the stress at the end of the increment ($\sigma_{n+1}$). For this reason, only the co-rotational part of the stress integration should be computed in UMAT as described above.

One major concern is the solution-dependent state variables. These variables are also passed in as the values at the beginning of the increment ($\varepsilon_{n}^{p}$ and ${\bar{\varepsilon }}_{n}^{p}$). However, the vector-valued or tensor-valued internal variables (e.g., $\varepsilon_{n}^{p}$) must be rotated to account for rigid body motion of the material in the increment. For this purpose, the rotation increment tensor (the increment of rigid body rotation of the element local co-rotational coordinate system) is also passed in so that the passed in vector- or tensor-valued internal variables are rotated appropriately in the UMAT subroutine (see [24] for the computation of the rotation increment tensor). Thereafter, the state variables must be updated based on the constitutive behavior to their values at the end of the increment ($\varepsilon_{n+1}^{p}$ and ${\bar{\varepsilon }}_{n+1}^{p}$).

## 4. Representative Applications

The previously described formulation is implemented into ABAQUS/Implicit by means of the user-defined subroutine UMAT. In reference [35], the model is calibrated for carbon fibre reinforced polymer (CFRP) IM7/8552 carbon/epoxy using test data from experiments on UD laminates. Furthermore, the performance of the elasto–plastic model is verified and validated via the FE simulation of the characterization tests performed in reference [36].

In the following, two numerical examples at two different scales are presented in order to demonstrate the applicability and capability of the proposed development in the context of geometrical non-linear analysis of composites. The examples discussed in the sequel are: (i) micro-buckling of UD composites subjected to compressive loading, and (ii) structural application involving laminated composites cylinder with free edges subjected to a point load. In these examples, mesh and time step convergence studies are carried out to ensure the validity of the results. In the time step convergence study, in each study, the maximum step size is controlled.

### 4.1. Micro-Buckling

**Figure 1:**
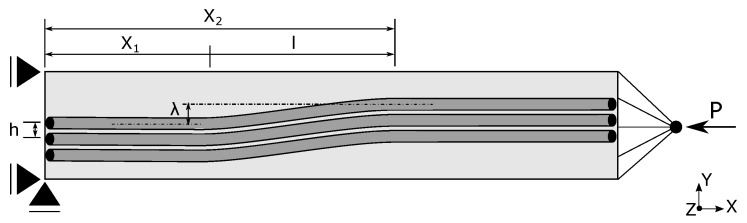
Schematic representation of fibre waviness, loading and boundary conditions.

To assess the capabilities of the current model at micro-scale, the failure under axial compression of unidirectional glass fiber reinforced polymers (GFRP) E-Glass/MY750 glass/epoxy ply with 60% fibre volume fraction is considered. Fibres naturally show a sinusoidal misalignment in continuous unidirectional fibre reinforced polymers [37] resulting in geometrical non-linearities. These geometrical non-linearities needed to be considered in the modeling, along with obvious material non-linearities, as this defines the accurate prediction of the UD compressive behavior. The schematic representation of the fibre waviness in the model is shown in Figure 1 along with its boundary and loading conditions. The model is a 3D homogenized representation of a layer of 15 glass fibres from a unidirectional ply to show the effect of misaligned fibres on the failure under compression, termed as kinking or micro-buckling in literature [38]. Overall fibre lengths of 500 μm are modeled, whereas width and thickness of the model come naturally from the fibre volume fraction and the number of fibres considered and are 93.75 μm and 6.25 μm, respectively. It should be noted that for the prediction of the different competing mechanisms leading to final kinking failure under compression, the micro-mechanical approaches with separate fibre-matrix modeling are useful [38]. However, the global stress-strain response can accurately be obtained through the current approach, with the advantages of significantly easier modeling and higher degree of computational efficiency.

The geometrical non-linearity of the fibres is introduced as in-plane sinusoidal angular misalignment in the model following [39] to initiate a kink band. The sinus waviness is over a length of 85 μm in the central region of the model with variable amplitudes, starting with an amplitude at one end and decreasing smoothly to an amplitude of 0 at the other end of the region bounded axially by $x_{1}\leq x\leq x_{2}$ in global x-direction as plotted in Figure 1. The fibre misalignment function is given below:

(57) $$y=\left\{\begin{array}{c} (i-1)h\;\;\;\;\;x<x_{1}\\ \left(i-1\right)h+\lambda \left(1-\frac{i}{N}\right)\left(1-cos\frac{\pi }{l}x\right)\;\;\;\;x_{1}\leq x\leq x_{2}\\ \left(i-1\right)h+2\lambda \left(1-\frac{i}{N}\right)\;\;\;\;\;x>x_{2}, \end{array}\right.$$

where *N* is the number of fibres in thickness of the layer, *h* refers to the distance between the center of adjacent fibres, *l* denotes the half wavelength, λ is the maximum value of amplitude, and $x_{1}$ and $x_{2}$ are the starting and ending positions of the waviness region, respectively.

A 3D finite element analysis (FEA) is performed to highlight the necessity of accounting for geometrical non-linearities at micro-level in the simulation of compressive failure of FRPs and to show the gained advantage of reduced computational costs through a homogenized modeling approach. The FE discretization consists of 9600 second-order, structured topology (3D 20-node brick elements—C3D20R).

The left face of the model is bounded in-plane i.e., global x- and y-axis, and the bottom left edge is bounded out-of-plane i.e., global z-axis. The right face of the model is coupled with a reference node through kinematic coupling, and axial force load is applied in negative x-direction. Since the kinking failure of unidirectional FRPs show a snap-back behavior, the riks method is used to capture the equilibrium path beyond limit points.

The material data needed for the model calibration are taken from reference [40]. The elastic material properties are reported in Table 1. Beside the elastic material constants, utilizing Equations (23)–(26), the yield function parameters $\zeta_{i}$ (i=1,4) that characterize the onset of yielding are listed in Table 2. Furthermore, the plastic potential function parameters $\varsigma_{i}$ (i=1,3) are provided in Table 3. These values are determined based on the plastic Poisson’s ratio $\nu_{23}^{p}=0.4$ and plastic distortion ratio $\mu_{12}^{p}\;=\;1.0$. Due to the lack of experimental data concerning the transverse shear, reasonable assumptions were made for transverse shear behavior.

**Figure 2:**
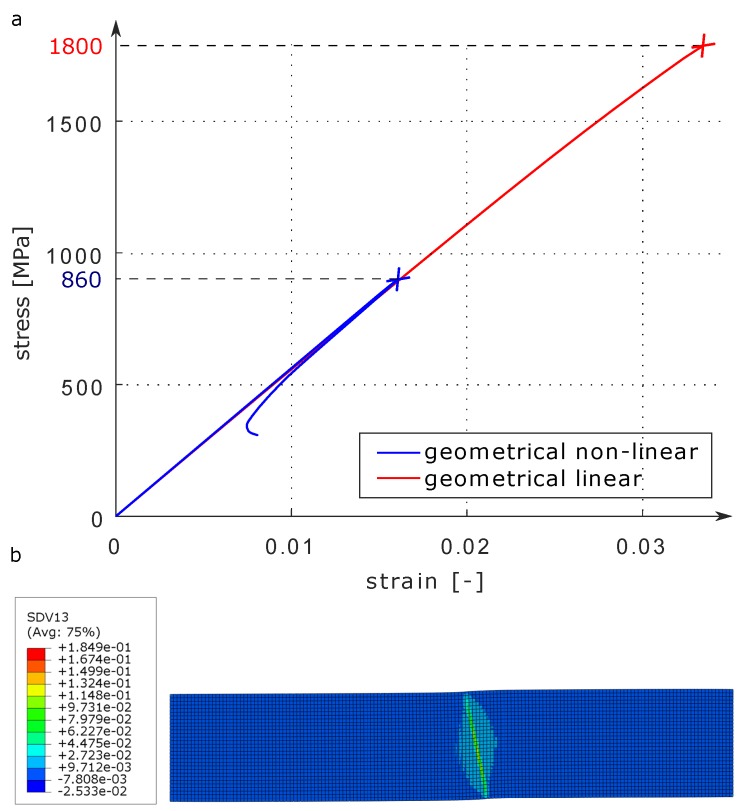
Axial compression response: (**a**) comparison of the results obtained by geometrical linear and co-rotational framework based geometrical non-linear solution and (**b**) kink band formation represented by the equivalent plastic strain (SDV).

The results in Figure 2a show the axial compression response curve for the geometrically linear and non-linear cases. In the plot, the axial stresses are calculated by taking the ratio of the applied incremental load with the initial cross-sectional area. Whereas, the strains are calculated by the ratio of the axial end shortening to the initial micro-model length. Under the applied compressive load, the shear stress concentrates at the misalignment region resulting in shear yielding and a sudden drop in load carrying capacity because of the instability which is seen as snap-back in the equilibrium path. This point of instability corresponds to the peak load. The shear localization, in turn, rotates the already misaligned region and forms the so-called kink band. The kink band formation represented by the equivalent plastic strain is depicted in Figure 2b. For the E-Glass/MY750 material, the calculated compressive strength through geometrically non-linear analysis is 860 MPa whereas the measured strength according to reference [40] is 800 MPa. On the other hand, the geometrical linear analysis with the same parameters shows an unrealistically high strength value of 1800 MPa. Considering the stochastic nature of compressive strength and limited experimental data available, it can be concluded that the current formulation is able to predict the compressive behavior reasonably well. Another thing to note is the highly reduced numerical size of the problem along with simpler modeling due to the homogenized material representation.

Using the micro-mechanical modeling approach where fibres and matrix are modeled separately, the detailed mechanism of the compressive failure mode can be investigated and observed, see reference [38]. Employing both the micro-mechanical and the current homogenized approach shows the same qualitative global response. However, the current approach is much more numerically efficient as compared to the micro-mechanical approach. For example, for the same model dimensions, the micro-mechanical approach in reference [38] required 20 times more elements for FE discretization. It should be noted that a direct quantitative comparison of the results obtained employing the presented approach with the micro-mechanical approach in reference [38] is not possible here since the materials investigated are different.

### 4.2. Laminated Composites Cylinder under Point Loads

**Figure 3:**
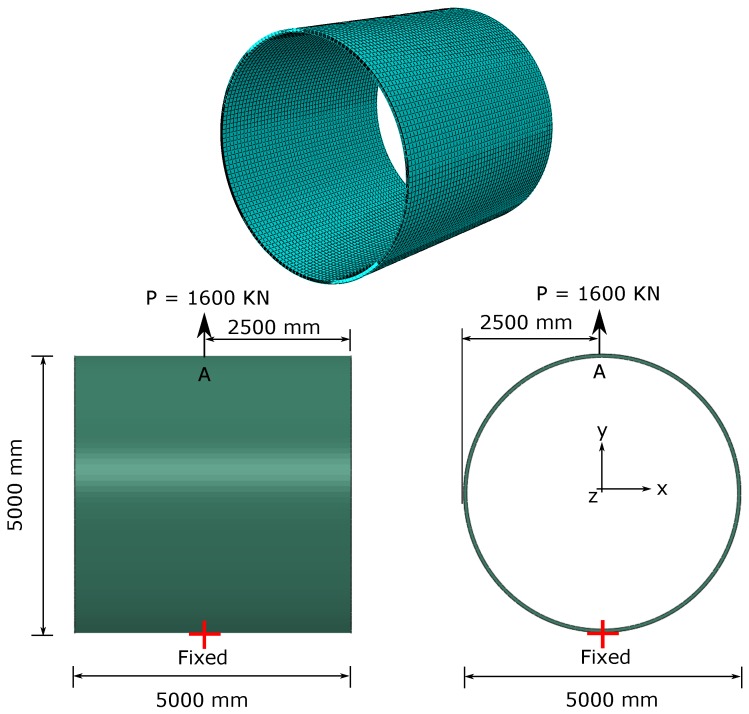
Laminated composites cylinder: geometric description, finite element (FE) mesh, boundary conditions and loading.

Herein, elasto–plastic co-rotational framework-based geometrical non-linear analysis of a cross-ply ${\left[0/90\right]}_{s}$ IM7/8551-7 carbon/epoxy laminated cylinder with free edges subjected to two opposite point loads is presented. The geometric description of the cylinder, FE mesh, boundary conditions and loading are depicted in Figure 3. The dimensions of the cylinder are: (i) length *L* = 5000 mm, (ii) mid-surface radius *R* = 2470 mm, and (iii) thickness *t* = 60 mm.

The elastic and plastic material properties needed for model calibration are given in Table 4, Table 5 and Table 6. Herein, the plastic Poisson’s ratio $\nu_{23}^{p}=0.5$ and plastic distortion ratio $\mu_{12}^{p}=1.0$. The 20-node quadratic brick element type C3D20R is used. After mesh convergence study, 31,600 elements are generated.

The load level and laminate stacking sequence are selected so that the strains remain small. The occurrence of material failure is checked by the invariant-based pressure-dependent quadratic asymmetric failure criteria (IQC) proposed in references [7,14].

**Figure 4:**
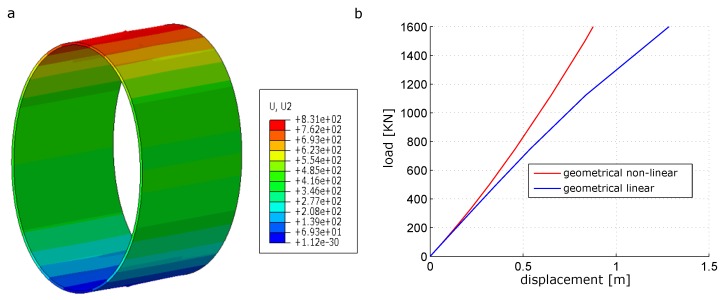
Laminated composites cylinder: (**a**) deformed configuration with $u_{2}$ and (**b**) loaddisplacement diagram.

The deformed configuration of the analyzed cylinder is shown in Figure 4a. The load-displacement diagrams at point A (directly under the load) is depicted in Figure 4b.

To point out the significance of including the geometrical non-linear effects, a geometrical linear elasto–plastic analysis is performed and the load-displacement diagram at point A is added to Figure 4. By comparing the load-displacement diagrams in Figure 4 obtained from the geometrical non-linear and geometrical linear analysis, a significant difference in the response of the structure under the same load level is observed. At point A, under the applied load, geometrically non-linear analysis resulted in a deflection of about is 0.83 m whereas the geometrical linear analysis under the same load level resulted in a deflection value of about 1.35 m.

**Figure 5:**
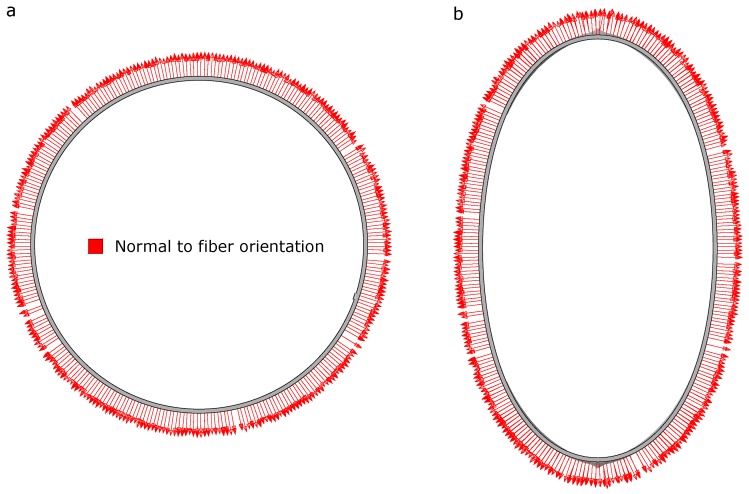
Fibre orientation: (**a**) initial configuration and (**b**) deformed configuration.

In Figure 5 the fibre orientation (represented by the normal to the nominal fibre orientation) change in the outer ply predicted by the geometrical non-linear analysis is depicted. In this graph, a significant variation of the fibre direction throughout the process is estimated. This fact stems from the large displacements and rotations experienced by the cylinder, which can notably affect the performance in service and cannot be captured using a geometrically linear model. This becomes evident in the current example and highlights the necessity of triggering the evolution of the fibre orientation along the deformation process. This issue can be only performed using a geometrically non-linear setting.

## 5. Conclusions

This paper was focused on the co-rotational formulation of an invariant-based anisotropic elasto–plastic model including detailed aspects of its numerical treatment and implementation in the finite element (FE) framework for geometrically non-linear analysis of fibre reinforced polymers (FRPs).

The proposed plasticity formulation assumed a pressure-dependent yield surface and a non-associate flow rule to capture realistic evolution of the inelastic behavior. In comparison to the yield function definition in [15], herein a new definition of the yield function that eases the calibration procedure and reduces the experimental effort was proposed. Hence, explicit expressions for the determination of the model parameters were provided.

On the computational side, the full computational algorithm of the proposed model was developed. Locally, the integration of the model evolution equations was given. Therein, explicit expressions necessary for the algorithmic computation of the model variables were provided. Globally, the consistent algorithmic tangent moduli was derived. Moreover, the important aspects of the model implementation in the general purpose FE code ABAQUS/Implicit were discussed.

Finally, two numerical examples at two different scales were presented pointing out the relevance of including the geometrical non-linear effects in the finite element analysis of FRPs. One key aspect was the possibility to allow for finite fibre rotation concurrently with the deformation process and thus the change of the material orientation.

The development of realistic models for complex materials usually requires a combination of more than one basic dissipative phenomena. Quasi-brittle materials, like FRPs, show damage and plasticity at the same time. Therefore, coupling the proposed plasticity anisotropic formulation with damage in order to describe the interaction between these processes represents the upcoming research focus. Among the different available options for damage modeling, those associated with kinematic enrichment of the FE mesh represent an appealing candidate. 

## Figures and Tables

**Table 1:** GFRP E-glass/MY750: elastic properties.

$E_{11}$ (MPa)	$E_{22}$ (MPa)	$G_{12}$ (MPa)	$\nu_{12}$	$\nu_{23}$
55,000	45,600	16,200	0.0987	0.40

**Table 2:** GFRP E-Glass/MY750: yielding parameters $\zeta_{i}$ at the onset of yielding.

$\zeta_{1}$	$\zeta_{2}$	$\zeta_{3}$	$\zeta_{4}$
0.00261641	0.00189036	0.0112808	0.000163349

**Table 3:** GFRP E-Glass/MY750: plastic potential parameters $\varsigma_{i}$.

$\varsigma_{1}$	$\varsigma_{2}$	$\varsigma_{3}$
1.0	1.0	−0.1071428

**Table 4:** Carbon fibre reinforced polymer (CFRP) IM7/8551-7: elastic properties.

$E_{11}$ (MPa)	c$E_{22}$ (MPa)	$G_{12}$ (MPa)	$\nu_{12}$	$\nu_{23}$
165,000	8400	5600	0.0173	0.50

**Table 5:** CFRP IM7/8551-7: yielding parameters $\zeta_{i}$ at the onset of yielding.

$\zeta_{1}$	$\zeta_{2}$	$\zeta_{3}$	$\zeta_{4}$
0.00176541	0.00127551	0.00926641	0.000110219

**Table 6:** CFRP IM7/8551-7: plastic potential parameters $\varsigma_{i}$.

$\varsigma_{1}$	$\varsigma_{2}$	$\varsigma_{3}$
1.0	1.0	−0.08333333
